# Impact of the COVID-19 Pandemic on Exercise Physiology Services in Australia: A Retrospective Audit

**DOI:** 10.1186/s40798-022-00483-2

**Published:** 2022-07-22

**Authors:** Patrick J. Owen, Shelley E. Keating, Christopher D. Askew, Kelly M. Clanchy, Paul Jansons, Ralph Maddison, Andrew Maiorana, Jenna McVicar, Suzanne Robinson, Niamh L. Mundell

**Affiliations:** 1 Institute for Physical Activity and Nutrition, School of Exercise and Nutrition Sciences, Deakin University, Geelong, VIC Australia; 2 School of Human Movement and Nutrition Sciences, The University of Queensland, Saint Lucia, QLD Australia; 3 VasoActive Research Group, School of Health and Behavioural Sciences, University of the Sunshine Coast, Sippy Downs, QLD Australia; 4 Sunshine Cost Health Institute, Sunshine Coast Hospital and Health Service, Birtinya, QLD Australia; 5 School of Health Sciences and Social Work, Griffith University, Gold Coast, QLD Australia; 6 Menzies Health Institute, Griffith University, Gold Coast, QLD Australia; 7 Department of Medicine, School of Clinical Sciences at Monash Health, Monash University, Clayton, VIC Australia; 8 Allied Health Department, Fiona Stanley Hospital, Perth, WA Australia; 9 School of Allied Health, Curtin University, Perth, WA Australia; 10 Curtin School of Population Health, Curtin University, Perth, WA Australia; 11 Deakin Health Economics, Institute for Health Transformation, Deakin University, Geelong, VIC Australia

**Keywords:** Telehealth, Coronavirus, Telemedicine, eHealth, mHealth, Rehabilitation

## Abstract

**Introduction:**

The COVID-19 pandemic has led to a shift in healthcare towards telehealth delivery, which presents challenges for exercise physiology services. We aimed to examine the impact of the COVID-19 pandemic on the reach, efficacy, adoption and implementation of telehealth delivery for exercise physiology services by comparing Australian practises before (prior to 25 January 2020) and during the COVID-19 pandemic (after 25 January 2020).

**Methods:**

This retrospective audit included 80 accredited exercise physiology clinicians. We examined relevant dimensions of the RE-AIM framework (reach, effectiveness, adoption and implementation) from the clinician perspective.

**Results:**

During the COVID-19 pandemic, 91% (*n* = 73/80) of surveyed clinicians offered telehealth delivery service, compared to 25% (*n* = 20/80) prior. Mean (SD) telehealth delivery per week doubled from 5 (7) to 10 (8) hours. In-person delivery decreased from 23 (11) to 15 (11) hours per week. Typical reasons for not offering telehealth delivery were client physical/cognitive incapacity (*n* = 33/80, 41%) and safety (*n* = 24/80, 30%). Clinician-reported reasons for typical clients not adopting telehealth delivery were personal preference (*n* = 57/71, 80%), physical capacity (*n* = 35/71, 49%) and access to reliable delivery platforms (*n* = 27/71, 38%). Zoom (*n* = 54/71, 76%) and telephone (*n* = 53/71, 75%) were the most commonly used platforms. Of the reasons contributing to incomplete treatment, lack of confidence in delivery mode was sevenfold higher for telehealth compared to in-person delivery. No serious treatment-related adverse events were reported.

**Conclusions:**

During the COVID-19 pandemic, telehealth delivery of exercise physiology services increased and in-person delivery decreased, which suggests the profession was adaptable and agile. However, further research determining comparative efficacy and cost-effectiveness is warranted.

**Supplementary Information:**

The online version contains supplementary material available at 10.1186/s40798-022-00483-2.

## Key Points

- During the COVID-19 pandemic, telehealth delivery of exercise physiology services increased and in-person delivery decreased.
- Clinician reasons for not offering telehealth delivery included client physical/cognitive incapacity and safety.
- Client reasons for not accepted telehealth delivery when offered included personal preference, physical capacity and access to reliable delivery platforms.

## Introduction

The coronavirus disease 2019 (COVID-19) pandemic continues to provide unprecedented challenges for healthcare systems worldwide. The imposition of infection control measures, such as physical distancing, has been essential for controlling transmission of COVID-19 [1]; however, these measures represent novel challenges for the delivery of in-person healthcare [2]. Telehealth delivery has emerged as a viable option to overcome these challenges and is conceptually defined as remote healthcare service engagement between the practitioner and client either synchronously (real-time interaction) or asynchronously (delayed interaction) [2].

The interest and demand for telehealth delivery of healthcare in general has markedly increased worldwide due to the COVID-19 pandemic [3]. For example, telehealth delivery of general outpatient care in the USA increased approximately 29-fold from October 2019 (pre-COVID-19 pandemic) to April 2020 (during the COVID-19 pandemic) [4]. Similarly, an approximately 24-fold increase in telehealth delivery of outpatient care was reported in an Australian hospital from February 2020 (pre-COVID-19 pandemic) to April 2020 (during the COVID-19 pandemic) [5]. Burgeoning evidence from a sample of 827 allied health professionals reported that despite two-thirds of clinicians not using telehealth delivery before the COVID-19 pandemic, the median proportion of clients engaged in services via telehealth delivery increased from 0% (pre-COVID-19 pandemic) to 60% (during the COVID-19 pandemic) [2]. Notably, the majority (82%) of participants in the study were physiotherapists and < 5% were exercise physiologists [2]; thus, these observations may not appropriately represent the broad spectrum of allied health professions.

Exercise physiology is one of the many allied health professions impacted by the changing healthcare landscape during the COVID-19 pandemic, and the adoption of telehealth delivery (also known as ‘tele-exercise’) [6] has potentially allowed for continued service provision. Within Australia, this was supported with policy changes in the form of new and revised industry professional standards [7], compensable service scheme recognition (e.g. WorkSafe Victoria [8], Victorian Traffic and Accident Commission [9]) and Australian Government funding of Medicare items for exercise physiology services [7]. To date, only one study of 72 Australian exercise physiology clinicians has examined the use of telehealth delivery borne from the COVID-19 pandemic, yet analyses were descriptive rather than inferential, which limited the capacity to draw conclusions [10]. Therefore, we aimed to determine the impact of the COVID-19 pandemic on the reach, efficacy, adoption and implementation of telehealth delivery for exercise physiology services by comparing Australian practises before (prior to 25 January 2020) and during the COVID-19 pandemic (after 25 January 2020; date of first confirmed case in Australia). We hypothesised that during the COVID-19 pandemic telehealth delivery of exercise physiology services would increase, whereas in-person delivery would decrease.

## Methods

### Study Design and Setting

A retrospective audit of exercise physiology services in Australia before (prior to 25 January 2020) and during the COVID-19 pandemic (after 25 January 2020) was conducted. The data collection period spanned 22 July 2020 to 10 November 2020 and included 80 accredited exercise physiology clinicians. The study was conducted in line with the National Statement on Ethical Conduct in Human Research (2007) and approved by Deakin University Human Ethics Advisory Group–Health (90–2020-200512). All participants provided written informed consent prior to involvement in the study.

### Participants

Participants were accredited exercise physiology clinicians currently practising within Australia during the COVID-19 pandemic (after 25 January 2020). Exercise physiologists are tertiary qualified allied health professionals who specialise in the prescription of exercise for a range of chronic conditions and are accredited by the peak exercise body within Australia (Exercise and Sports Science Australia) [11]. Potential participants were primarily sought through social media advertisement (e.g. Twitter and Facebook), newsletters and magazines periodically released by the professional accreditation body, as well as via word-of-mouth through the professional networks of the study authors.

### Data Collection

Data were collected via an anonymous online survey (Qualtrics, Provo, Utah, USA; Additional file 1: Data S1) of clinicians only (i.e. no clients were surveyed and client-based questions were reported from the perception of the clinician upon reflecting on their overall client load). We collected data regarding: (1) demographics (clinicians and their clients), (2) usual practise (prior to 25 January 2020) and (3) changes to practise (after 25 January 2020). The implementation of telehealth delivery was a focal point of our investigation into changes in practice, and we therefore adopted relevant dimensions of the RE-AIM framework (i.e. reach, effectiveness, adoption and implementation) from the clinician perspective [12]. RE-AIM has been successfully applied to understand the relative strengths and weaknesses of different approaches to chronic disease self-management including in-person counselling, group education classes, telephone counselling and internet resources [13]. A summary of each RE-AIM domain and reported outcomes is presented in Table 1. In brief, reach included practice type, geographical location, session frequency, duration and cost, client demographics and barriers to offering telehealth. Adoption included number of clinicians and hours of service provision via telehealth delivery. Implementation included method of delivery (asynchronously: delayed interaction, e.g. email; synchronously: real-time interaction, e.g. video calls), service and platform type, supporting resources, barriers and treatment completion rate. The retrospective design of the current study precluded robust estimates of effectiveness, and therefore, only safety (treatment- and non-treatment-related serious and non-serious adverse events) was considered for this domain. Serious adverse events were defined as any untoward medical occurrent that results in death, is life-threatening or requires hospitalisation. Non-serious adverse events were defined as any other untoward medical occurrent. Adverse events were classified as treatment related if they were definitely, possibly or probably related to the provision of exercise physiology services. Maintenance (i.e. integration into usual care and efficacy after six or more months) was also not considered due to the retrospective design of the current study.

**Table 1:** Domains of the RE-AIM framework and relevant outcomes reported in the current study. Adapted from the RE-AIM framework [12, 13]

Domain	Outcome
Reach	Clinician role Practice geographical location and type Time per exercise physiology session Financial cost per exercise physiology session Client demographics: age, body mass index, geographical location, referral source and medical conditions Frequency of exercise physiology service provision Barriers relating to offering telehealth to clients
Efficacy	Treatment-related serious adverse events: any untoward medical occurrence associated with treatment that results in death is life-threatening or requires hospitalisation Non-treatment-related serious adverse events: any untoward medical occurrence not associated with treatment that results in death is life-threatening or requires hospitalisation Treatment-related non-serious adverse events: any untoward medical occurrence associated with treatment that is not deemed a serious adverse event Non-treatment non-serious adverse events: any untoward medical occurrence not associated with treatment that is not deemed a serious adverse event
Adoption	Number of clinicians that provided services Service delivery hours per week per clinician
Implementation	Delivery method utilised: asynchronous/synchronous Delivery platforms utilised Services provided Client hardware preferences Additional resources utilised Barriers relating to client acceptance Rate of treatment completion
Maintenance	Not evaluated

The following telehealth delivery platforms were considered: Attend Anywhere (www.attendanywhere.com), Cliniko (www.cliniko.com), Coviu (www.coviu.com), Doxy.me (www.doxy.me), Facebook Messenger (www.messenger.com), FaceTime (www.apps.apple.com/us/app/facetime/id1110145091), HealthBank (www.healthbank.io), HealthConnect (www.virtusa.com/solutions/healthconnect), Healthdirect Video Call (vcc.healthdirect.org.au), Microsoft Teams (www.microsoft.com/en-au/microsoft-teams), MyPhysioRehab (www.myphysiorehab.com), Pexip (www.pexip.com), Physitrack (www.physitrack.com), PracMan (www.pracman.com.au), Skype (www.skype.com), Trainerize (www.trainerize.com), Vidyo (www.vidyo.com), WhatsApp (www.whatsapp.com), Zoom (www.zoom.us), telephone.

### Statistical Analyses

All analyses were conducted using Stata (17, StataCorp, College Station, TX). Differences between in-person and telehealth delivery were assessed by chi-square test. Odds ratios (OR) and corresponding 95%CI for clinicians offering telehealth delivery (yes/no) based on explanatory variables (clinician, practice and client demographical details) were determined via penalised maximum likelihood logistic regression [14, 15]. Univariate associations between percentage of clients offered and clients who accepted telehealth delivery, and explanatory variables were assessed via linear regression. Sensitivity analyses employed the false discovery rate adjustment [16]. An alpha of 0.05 was adopted for all analyses.

## Results

### Reach

The total number of clinicians offered this survey was not measured given the breadth of dissemination. However, when considering recent industry estimates [17] of 6315 accredited exercise physiologists as an upper limit, the current study reached 1.3% (*n* = 80/6, 315) of the active workforce.

Clinician employment demographics are shown in Table 2. Mean (SD) duration of practise as an accredited exercise physiologist was 7 (6) years (min: 0.5 years, max: 29 years). Approximately half (*n* = 39/80, 49%) of clinician practices were located in the state of Victoria. No clinicians from Tasmania, Northern Territory or Australian Capital Territory provided data. Approximately half (*n* = 38/80, 48%) of clinicians were employed by private practices in salary roles. Mean (SD) duration of an exercise physiology session, irrespective of the COVID-19 pandemic, was 51 (21) min (min: 20 min, max: 150 min) and incurred an hourly fee of A$111.57 (A$46.61; min: A$10.00, max: A$195.00).

**Table 2:** Employment demographics reported by the 80 accredited exercise physiology clinicians currently practising within Australia during the COVID-19 pandemic (after 25 January 2020)

Variable	Clinicians (*n* = 80)
*Clinician role, n (%)*	
Salary (private clinics/companies)	38 (47.5)
Director	21 (26.3)
Contractor	13 (16.3)
Academic	4 (5.0)
Practice manager	2 (2.5)
Sole trader	2 (2.5)
*Practice geographical location, n (%)*	
Victoria	39 (48.8)
New South Wales	20 (25.0)
Western Australia	10 (12.5)
Queensland	9 (11.3)
South Australia	2 (2.5)
*Practice type, n (%)*	
Private clinic/company	38 (47.5)
Sole trader	13 (16.3)
Hospital	12 (15.0)
University clinic	12 (15.0)
Not for profit	4 (5.0)
Community health	1 (1.3)

Data are count (percentage within-group). No data within this table were missing

Demographics of the clients commonly treated by clinicians, irrespective of the COVID-19 pandemic, are presented in Additional file 1: Table S1. The majority of clinicians typically treated metropolitan-based clients aged 46–75 years with a body mass index of 25–34.9 kg/m^2^. The majority of clinicians reported receiving referral for musculoskeletal complaints (*n* = 60/80, 75%), type 2 diabetes (*n* = 50/80, 63%) and/or cardiovascular disease (*n* = 48/80, 60%). Clinicians also reported typically receiving the following referrals: paediatric healthcare (*n* = 2), pain management (*n* = 1), falls prevention (*n* = 1), general health and fitness (*n* = 1) and aged care (*n* = 1). Clinicians most commonly received Medicare (*n* = 49/80, 61%) and private referrals (*n* = 49/80, 61%), followed by those from the National Disability Insurance Scheme (NDIS [18]; *n* = 37/80, 46%). Other forms of typical client referral pathways included: Australian health practitioner/general practitioner (*n* = 1), student-led paid services (*n* = 1), home care packages (*n* = 1), self-funded (*n* = 1), residential (*n* = 1) and university-funded (*n* = 1).

During the COVID-19 pandemic, 91% (*n* = 73/80) of surveyed clinicians offered telehealth delivery to a mean (SD) of 49 (64) clients (min: 2 clients, max: 300 clients). On average, clinicians offered telehealth delivery to 85% of clients. Clinician reasons for not offering telehealth delivery are shown in Table 3. The most common reasons for not offering telehealth delivery were client physical/cognitive capacity (*n* = 33/80, 41%) and client safety (*n* = 24/80, 30%). Clinicians that had clients referred via any insurance scheme were less likely to offer telehealth delivery (OR [95%CI]: 0.09 [0.01, 0.99]). Clinicians that reported typically having clients with a body mass index of 35–39 kg/m^2^ (*β* = 0.16, *P* = 0.02) or obesity/metabolic condition (*β* = 0.14, *P* = 0.04) were more likely to offer telehealth delivery.

**Table 3:** Typical reasons for not offering telehealth delivery reported by the 80 accredited exercise physiology clinicians currently practising within Australia during the COVID-19 pandemic (after 25 January 2020)

Variable	Clinicians (*n* = 80)
Client physical/cognitive capacity	33 (41.3)
Client safety	24 (30.0)
Access to a reliable platform	15 (18.8)
Client language/cultural barriers	7 (8.8)
Ceased all services	6 (7.5)
Cost of set-up for client	3 (3.8)

Data are count (percentage within-group). No data within this table were missing

### Effectiveness (safety)

No treatment-related serious adverse events occurred in clients receiving telehealth or in-person delivery. No non-treatment-related serious adverse events occurred in clients receiving telehealth delivery. Three non-treatment-related serious adverse events occurred in clients receiving in-person delivery: COVID-19 diagnosis (*n* = 2), death from a pre-existing heart condition (*n* = 1). One treatment-related adverse event occurred during telehealth delivery involving a non-serious pacemaker issues. Two treatment-related non-serious adverse events occurred during in-person delivery: brief loss of consciousness (*n* = 1), fall (*n* = 1). One non-serious non-treatment-related fall occurred in a client receiving telehealth delivery. Five non-serious non-treatment-related adverse events occurred in clients receiving in-person delivery: dizziness (*n* = 3), seizure (*n* = 1), chest pain (*n* = 1).

### Adoption

During the COVID-19 pandemic, 89% (*n* = 71/80) of clinicians implemented telehealth delivery, compared to 25% (*n* = 20/80) pre-COVID-19 pandemic. In-person practice decreased from 99% (*n* = 79/80) pre-COVID-19 pandemic to 86% (*n* = 69/80) during the COVID-19 pandemic. Mean (SD) telehealth delivery hours per week per clinician more than doubled from 4.6 (7.1) hours pre-COVID-19 pandemic to 10.3 (7.8) hours during the COVID-19 pandemic. In contrast, in-person delivery hours per week per clinician decreased from 23.0 (10.6) hours pre-COVID-19 pandemic to 14.5 (11.1) hours during the COVID-19 pandemic.

### Implementation

**Fig. 1:**
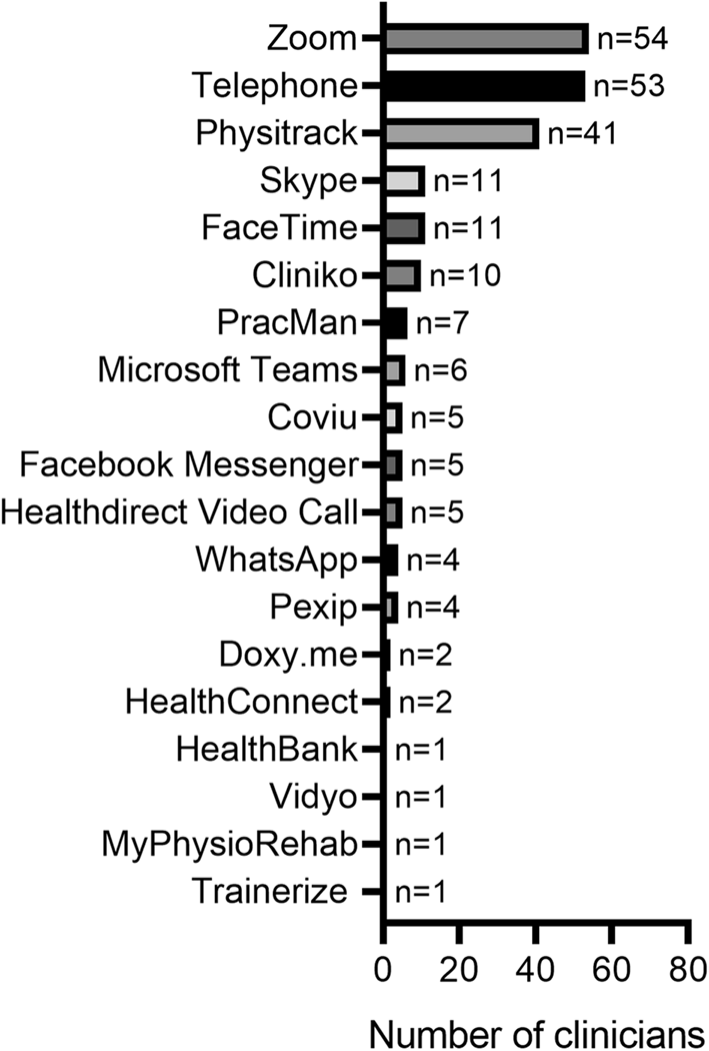
Telehealth delivery platforms used by the 80 accredited exercise physiology clinicians currently practising within Australia during the COVID-19 pandemic (after 25 January 2020)

**Fig. 2:**
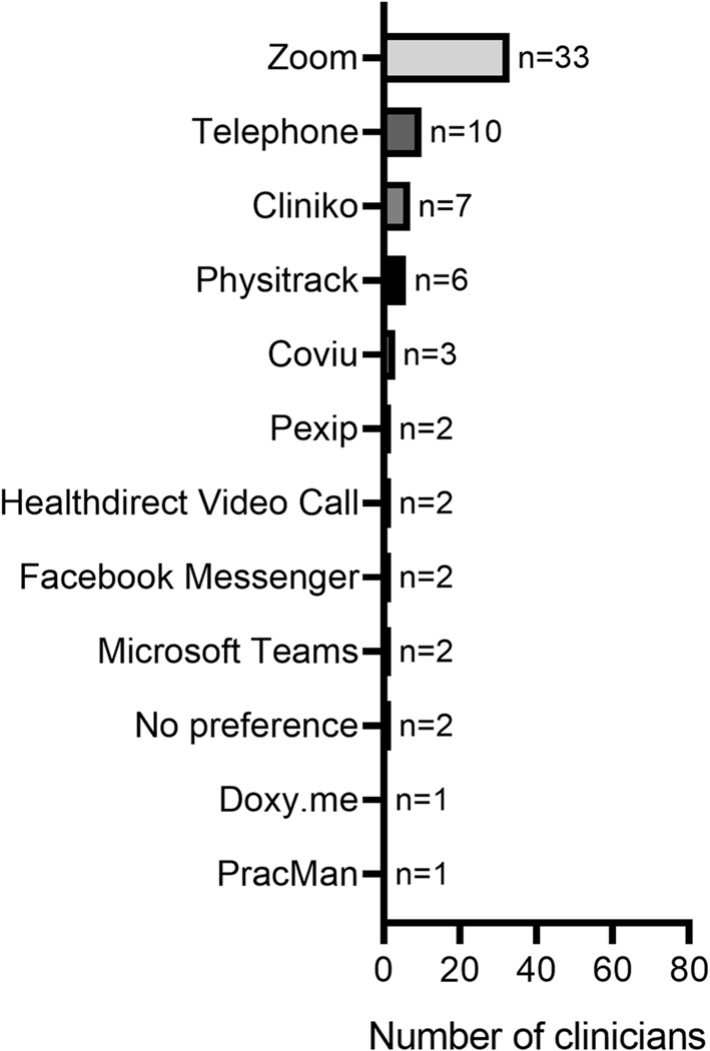
Telehealth delivery platforms rated the best by the 80 accredited exercise physiology clinicians currently practising within Australia during the COVID-19 pandemic (after 25 January 2020)

**Fig. 3:**
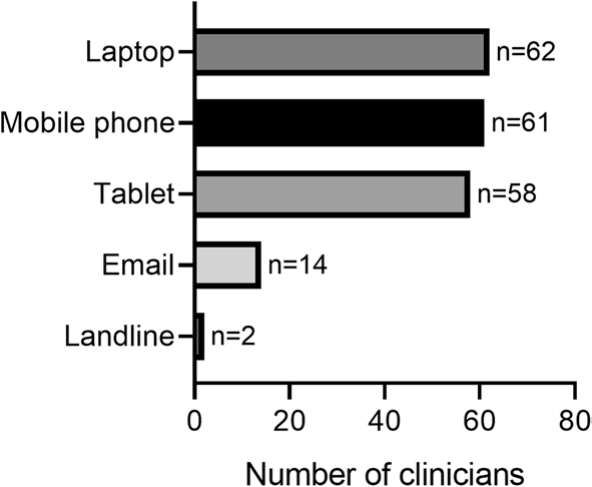
Telehealth delivery devices used by the clients reported by the 80 accredited exercise physiology clinicians currently practising within Australia during the COVID-19 pandemic (after 25 January 2020)

Of the clinicians that implemented telehealth delivery, the majority utilised synchronous (*n* = 68/71, 96%) rather than asynchronous methods (*n* = 28/71, 39%). Education (*n* = 39/71, 55%) and counselling (*n* = 37/71, 52%) were also commonly implemented as part of telehealth delivery. Zoom (*n* = 54/71, 76%), telephone (*n* = 53/71, 75%) and Physitrack (*n* = 41/71, 58%) were the most commonly used delivery platforms by clinicians (Fig. 1). Zoom was considered the best telehealth delivery platform by 46% (*n* = 33/71) of clinicians that utilised telehealth delivery (Fig. 2). The majority of clinicians reported that their typical clients used laptops (*n* = 62/71, 87%), mobile phones (*n* = 61/71, 86%) and tablets (*n* = 58/71, 82%) for telehealth delivery (Fig. 3).

Additional resources utilised by clinicians to facilitate telehealth delivery are shown in Table 4. Notably, 37% (*n* = 26/71) of clinicians that implemented telehealth delivery undertook additional professional development. Mean (SD) professional development duration was 3.4 (2.4) hours (min: 1 h, max: 10 h).

**Table 4:** Additional resources used to facilitate telehealth delivery reported by the 80 accredited exercise physiology clinicians currently practising within Australia during the COVID-19 pandemic (after 25 January 2020)

Variable	Clinicians that delivered telehealth (*n* = 71)
Text message reminders	51 (71.8)
Follow-up phone calls	46 (64.8)
Written instructions	46 (64.8)
Apps for a smart phone or tablet	39 (54.9)
Educational material on the condition/issue	35 (49.3)
Videos	35 (49.3)
Provision/purchase of equipment or devices	30 (42.3)
Logbooks and diaries	26 (36.6)
Suggested websites for further information	20 (28.2)
Diagrams or booklets	18 (25.4)
Other	2 (2.8)

Data are count (percentage within-group). No data within this table were missing

Collectively, 52% of clients accepted the offer to use telehealth delivery for exercise physiology services. Reasons for not accepting and non-completion of treatment for telehealth delivery and in-person services are reported in Table 5. Clinicians reported the most typical client reasons for not accepting telehealth delivery were personal preference (*n* = 57/71, 80%), physical capacity (*n* = 35/71, 49%) and access to a reliable platform (*n* = 27/71, 38%). Clients aged 36–45 years (*β* = 0.21, *P* = 0.001) and those who were referred via Medicare (*β* = 0.14, *P* = 0.04) were more likely to accept telehealth delivery. Among clients who accepted telehealth delivery, 75% completed treatment. When compared to in-person delivery, clients receiving care via telehealth delivery were 6.7 times more likely (*P* < 0.001) to not complete treatment due to a lack of confidence in the delivery mode, 3.9 times more likely (*P* = 0.005) to not complete the treatment course due to a lack of understanding of telehealth delivery and 3.1 times more likely (*P* < 0.001) to not complete the treatment course due to a lack of interest or lack of perceived importance of telehealth delivery. Conversely, clients receiving in-person delivery were 6.2 times more likely (*P* < 0.001) to not complete treatment due to safety concerns borne from COVID-19 when compared to those using telehealth delivery. Following sensitivity analyses applying the false discovery rate adjustment, statistical significance remained (Additional file 1: Table S2).

**Table 5:** Typical client reasons for not accepting or completing treatment reported by the 80 accredited exercise physiology clinicians currently practising within Australia during the COVID-19 pandemic (after 25 January 2020)

Variable	Clinicians that delivered telehealth (*n* = 71)	Clinicians that delivered in-person (*n* = 69)
*Client non-acceptance, n (%)*		
Client preference of delivery mode	57 (80.3)	–
Client not suited due to physical abilities	35 (49.3)	–
Client or clinician unable to access a reliable platform	27 (38.0)	–
Client or clinician cost of set-up	14 (19.7)	–
Client language or cultural barriers	9 (12.7)	–
Client safety	7 (9.9)	–
*Client non-completion, n (%)*		
Client preference of delivery mode	24 (33.8)	14 (20.3)
Client lack of confidence in delivery mode	**21 (29.6)**^‡^	3 (4.4)
Client lack of understanding in delivery mode	**16 (22.5)**^†^	4 (5.8)
Client lack of interest or importance in delivery mode	**29 (40.9)**^‡^	9 (13.0)
Client safety	**3 (4.2)**^‡^	18 (26.1)
Client or clinician unable to access reliable platform or venue	13 (18.3)	8 (11.6)
Problems unrelated to service delivery	16 (22.5)	14 (20.3)

Data are count (percentage within-group).

^‡^*P* < 0.001,^†^*P* = 0.005 compared to in-person (bold). No data within this table were missing

## Discussion

The results of the current study showed telehealth delivery of exercise physiology services increased and in-person delivery decreased in response to the COVID-19 pandemic (adoption). The study demonstrated that most clinicians implemented synchronous telehealth delivery (implementation) to a range of client demographics from varying referral pathways (reach). The primary reasons for not offering telehealth delivery were client physical/cognitive incapacity and safety concerns (reach). Approximately half the clients accepted an offer of telehealth delivery, with personal preference, physical/cognitive incapacity and access to reliable telehealth platforms cited by clinicians as the most common client reasons for non-acceptance (implementation). Clinicians reported that client non-compliance primarily stemmed from a lack of confidence, understanding and perceived importance of telehealth delivery (implementation). Finally, telehealth delivery appeared safe for the provision of exercise physiology services (effectiveness).

Clinicians in our study reported a marked shift from in-person to telehealth delivery of exercise physiology services due to the COVID-19 pandemic. This aligns with previous observations regarding telehealth delivery uptake due to COVID-19 among several allied health professions, including exercise physiology [10], as well as among a cohort of primarily physiotherapy allied health clinicians [2]. These findings suggest that allied health clinicians were readily able and willing to adapt practices to enable client access to their telehealth services. It is therefore logical to consider the comparative efficacy between these delivery modes. Given the marked heterogeneity in populations, interventions, comparators and outcomes, robust meta-analytical studies regarding comparative efficacy between telehealth and in-person delivered exercise training are sparce, although a meta-analysis of randomised controlled trials that examined telehealth and in-person delivery of exercise-based cardiac rehabilitation demonstrated comparable efficacy between the two modalities [19]. Notably, in addition to the limited number of studies included in the quantitative synthesis (*n* = 11), all trials were published and thus conducted in 2014 or earlier. Given myriad recent advancements in telehealth hardware, software and practise [20], studies investigating state-of-the-art telehealth delivery methods (e.g. artificial intelligence assisted) are warranted. Moreover, there is a need for trials that directly compare these modalities, rather than efficacy compared to control alone.

The current study identified several barriers regarding the implementation and delivery of telehealth exercise physiology services. Concerns about client physical/cognitive incapacity and safety were the most common reason for not offering telehealth delivery. Exercise physiologists were previously reported to have safety concerns when providing video consultations owing to falls risk and an inability to monitor environment and physiological responses during the provision of exercise training [10]. This was similarly observed in a cohort of allied health clinicians who primarily provided physiotherapy [2]. Moreover, clinicians in the current study reported that nearly half the clients shared concerns of their own physical/cognitive incapacity regarding telehealth delivery. This was markedly greater than the 17% of older adults identified in a systematic review of 56 studies that cited hand–eye coordination, visual acuity, mental acuity or auditory acuity as barriers to telehealth delivery of general care [21]. This highlights a potential barrier specific to telehealth delivery of exercise physiology that requires consideration by clinicians offering these services. Clinicians in our study also noted that key barriers to acceptance of telehealth delivery among clients were personal preference and access to reliable platforms. These observations align with a systematic review of barriers to telehealth that identified technical literacy (17%) and lack of desire (13%) as the most common barriers to telehealth delivery of care in older adults [21]. Collectively, these barriers provide insight into both client and clinician concerns associated with telehealth delivery of exercise physiology services and should be further explored before attempting widespread implementation. Failure to address these barriers may reduce the likelihood of more vulnerable clients, such as those with concerns regarding capacity, receiving exercise physiology services.

In the current study, almost half the clinicians reported clients who were federally funded by the NDIS to receive individualised financial packages of support for people with a permanent and significant disability under the age of 65 years. This is surprising based on the relatively recent expansion of claimable items for exercise physiology via NDIS. Overall, 2557 exercise physiology and physical wellbeing-approved service providers are registered to provide services via the NDIS [18]. Telehealth delivery can reduce barriers to healthcare access for individuals with a disability through providing lower costs of care, lower transportation costs, less exposure to communicable diseases especially during a pandemic and decreased need for paid personal assistance services [22, 23]. A survey of 2391 NDIS participants observed 63% changed NDIS-funded allied healthcare services to videoconference or telephone, with 7% receiving exercise physiology [24]. Our findings support the notion that the NDIS is a growing service provision area for exercise physiology.

The current study was strengthened by the broad ranging demographics of clients treated by the participating clinicians. This reflects the wide range of age, body mass index, geographical location, referral pathways and health conditions commonly encountered within the exercise physiology profession in Australia [25].

**Fig. 4:**
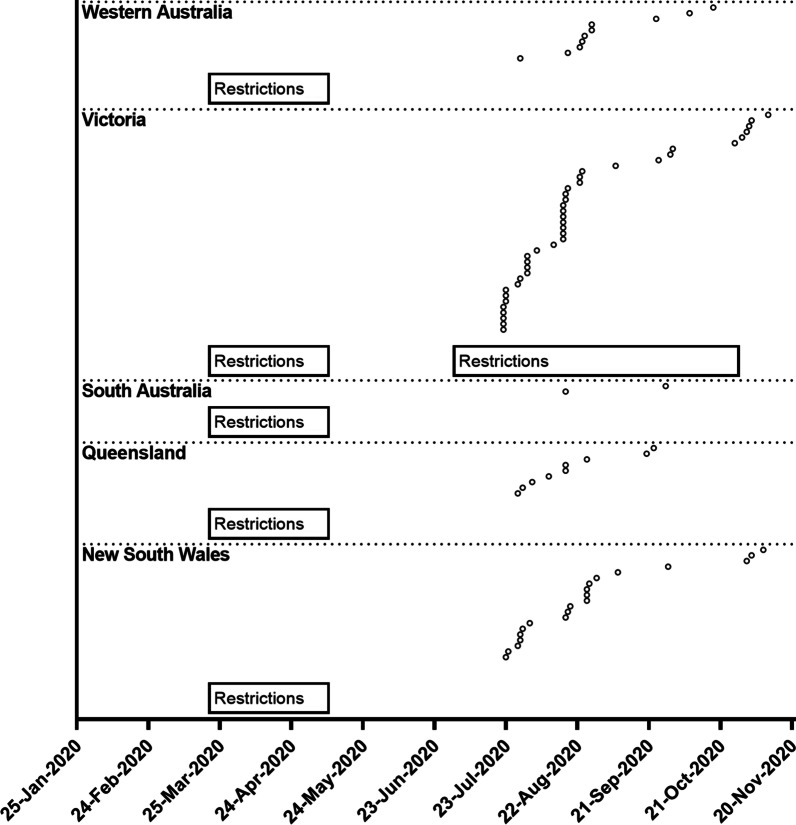
COVID-19 restriction and accredited exercise physiology clinician recruitment timelines by state. 25-Jan-2020: Date of first confirmed COVID-19 case in Australia

The study has several limitations that should be considered when interpreting results. First, participants only represented five of the eight Australian states and territories, with nearly half the sample from Victoria. Comparably, the governing body for exercise physiology in Australia reported 20% of members reside in Victoria [25]. This limits generalisability of findings to the omitted geographical locations. Second, generalisability may also be impacted by recruitment bias, whereby clinicians with a penchant for telehealth delivery were more likely to participate. Investigating similar questions posed in the current study with a random sample of clinicians is warranted. Third, government restrictions in response to the COVID-19 pandemic differed between states and territories. All clinicians in the current study were subject to national restrictions from 23 March 2020 to May 2020, whereas Victorian clinicians also had restrictions from 8 July 2020 to 26 October 2020 (Fig. 4). These shenanigans may in part explain the greater number of participants from Victoria, given the increased need to utilise telehealth delivery. Fourth, given the sample size of our study, conclusions regarding comparative safety between delivery modes should be interpreted with caution. Fifth, as data were collected retrospectively from clinicians, recall bias may have impacted reliability. Finally, as Australia is currently in the midst of the COVID-19 pandemic, we were unable to examine recovery trends post-COVID-19 pandemic. Future research should replicate the current study following the COVID-19 pandemic to allow for insight into the recovery of the exercise physiology profession, as well as the maintenance of telehealth delivery per the RE-AIM framework [12].

## Conclusion

During the COVID-19 pandemic, telehealth delivery services increased, and in-person services decreased among Australian accredited exercise physiology clinicians (adoption). Most clinicians implemented synchronous telehealth delivery (implementation) to a range of client demographics from varying referral pathways (reach). Common reasons for not offering telehealth delivery were physical/cognitive incapacity and safety concerns (reach), whereas client reasons for not accepting telehealth delivery were personal preference, physical/cognitive incapacity and access to reliable platforms (implementation). Client non-compliance appeared to stem from a lack of confidence and understanding of the perceived importance of telehealth delivery (implementation). Finally, telehealth delivery appeared safe (effectiveness). These data suggest that exercise physiology services in Australia can be adaptable and agile. However, further research is warranted to determine the comparative efficacy and cost-effectiveness between delivery modes.

## Supplementary Information

**Additional file1**: **Data S1**. Survey provided to the 80 accredited exercise physiology clinicians currently practising within Australia during the COVID-19 pandemic (after 25 January 2020). **Table S1**. Typical client demographics reported by the 80 accredited exercise physiology clinicians currently practising within Australia during the COVID-19 pandemic (after 25 January 2020). **Table S2.** Sensitivity analysis applying the false discovery rate adjustment to P-values from Table 5.

## Data Availability

Data available on request from the authors.
