# Supplementary material for: Impact of the COVID-19 Pandemic on Exercise Physiology Services in Australia: A Retrospective Audit
Source: Sports Med Open. 2022 Jul 22;8:94. doi: 10.1186/s40798-022-00483-2 (PMC9306237; doi:10.1186/s40798-022-00483-2)
Supplement: Supplementary file 1 — Additional file1: Data S1. Survey provided to the 80 accredited exercise physiology clinicians currently practising within Australia during the COVID-19 pandemic (after 25 January 2020). Table S1. Typical client demographics reported by the 80 accredited exercise physiology clinicians currently practising within Australia during the COVID-19 pandemic (after 25 January 2020). Table S2. Sensitivity analysis applying the false discovery rate adjustment to P-values from Table 5. [file 40798_2022_483_MOESM1_ESM.docx]

**Supplementary Table S1**. Typical client demographics reported by the 80 accredited exercise physiology clinicians currently practising within Australia during the COVID-19 pandemic (after 25 January 2020).

| Variable | Clinicians (n=80) |
| --- | --- |
| **Client age, n (%)** |  |
| 0-18 years | 11 (13.8) |
| 19-25 years | 20 (25.0) |
| 26-35 years | 27 (33.8) |
| 36-45 years | 29 (36.3) |
| 46-55 years | 45 (56.3) |
| 56-65 years | 52 (65.0) |
| 66-75 years | 52 (65.0) |
| 76-85 years | 35 (43.8) |
| >85 years | 17 (21.3) |
| **Client body mass index, n (%)** |  |
| <25 kg/m^2^ | 21 (26.3) |
| 25-29.9 kg/m^2^ | 57 (71.3) |
| 30-34.9 kg/m^2^ | 57 (71.3) |
| 35-39.9 kg/m^2^ | 37 (46.3) |
| 40-44.9 kg/m^2^ | 13 (16.3) |
| >45 kg/m^2^ | 6 (7.5) |
| **Client location, n (%)** |  |
| Metropolitan | 60 (75.0) |
| Regional | 26 (32.5) |
| Rural | 22 (27.5) |
| Remote | 9 (11.3) |
| **Client referral, n (%)** |  |
| Medicare | 49 (61.3) |
| Private | 49 (61.3) |
| National Disability Insurance Scheme | 37 (46.3) |
| Department of Veterans’ Affairs | 29 (36.3) |
| Workcover | 25 (31.3) |
| Other | 6 (7.5) |
| Hospital | 5 (6.3) |
| Transport Accident Commission | 4 (5.0) |
| Insurance | 2 (2.5) |
| **Client condition, n (%)** |  |
| Musculoskeletal | 60 (75.0) |
| Type 2 diabetes | 50 (62.5) |
| Cardiovascular | 48 (60.0) |
| Obesity/metabolic (non-type 2 diabetes) | 41 (51.3) |
| Mental health | 36 (45.0) |
| Neurological | 32 (40.0) |
| Cancer/post-cancer | 29 (36.3) |
| Pulmonary | 21 (26.3) |
| Renal | 6 (7.5) |
| Other | 6 (7.5) |
| Pre/post solid organ transplant | 1 (1.3) |

Data are count (percentage within-group). No data within this table were missing.

**Supplementary Table S2.** Sensitivity analysis applying the false discovery rate adjustment to P-values from Table 5.

| **Variable** | **Original P-value** | **Benjamini-Hochberg-adjusted P-value** |
| --- | --- | --- |
| **Client non-completion, n (%)** |  |  |
| Preference | 0.0722513518064423 | 0.101 |
| Confidence | **0.0000749527183003** | **0.001** |
| Understanding | **0.0046612417654260** | **0.008** |
| Interest | **0.0002170964413823** | **0.001** |
| Safety | **0.0002926340820943** | **0.001** |
| Access | 0.2658978590654710 | 0.310 |
| Unrelated | 0.7461633006476450 | 0.746 |

**Supplementary Data S1**. Survey provided to the 80 accredited exercise physiology clinicians currently practising within Australia during the COVID-19 pandemic (after 25 January 2020).

Please select the option that best describes your practice:

- Private clinic/company
- Sole trader
- University clinic
- Hospital
- Not for profit
- Other (please specify)

Please select the option that best describes your role:

- Clinician - salary
- Clinician - contractor
- Director
- Practice manager
- Other (please specify)

Please detail the number of years you have been practicing as an Accredited Exercise Physiologist:

After 25th Jan 2020, on average, how many hours per week would you spend delivering Exercise Physiology services face-to-face?

After 25th Jan 2020, on average, how many hours per week did you deliver Exercise Physiology services via Telehealth platforms?

Did you have to undertake any extra training in order to begin delivering Telehealth services to clients?

- No
- Yes

How many hours of extra training did you undertake in order to begin delivering Telehealth services to clients?

Please select the options that most closely represent the demographics of your overall caseload since the 25th of January 2020 (more than one answer allowed):

Age Range

- 0 - 18
- 19 - 25
- 26 - 35
- 36 - 45
- 46 - 55
- 56 - 65
- 66 - 75
- 76 - 85
- Over 85

BMI

- <25
- 25 - 29.9
- 30 - 34.9
- 35 - 39.9
- 40 - 44.9
- >45

Geographical Location

- Metropolitan
- Rural
- Remote
- Regional

Referral Type

- Medicare
- DVA
- Private
- NDIS
- Workcover
- Other (please specify)

Primary Presenting Conditions (Select all that apply)

- Obesity/metabolic (other than Type 2 diabetes)
- Type 2 diabetes
- Cardiovascular
- Neurological
- Pulmonary
- Renal
- Musculoskeletal
- Cancer/post-cancer
- Pre/post solid organ transplant
- Mental health
- Other (Please specify)

What were/are the main Exercise Physiology service components you provided using Telehealth platforms (more than one entry accepted)?

- Synchronous exercise sessions (AEP/client both present in real time during session)
- Asynchronous exercise sessions (AEP/client not both present in real time during session; e.g. unsupervised home-based exercise program)
- Education
- Health or behaviour change counselling
- Not applicable

What platforms did/do you use in order to undertake Exercise Physiology services via Telehealth? (Sort from most '1' to least '16' frequent; only rank those you use/used)

- Telephone
- Zoom
- Physitrack
- Subscribed practice management software
- Skype
- Microsoft Teams
- Cliniko
- Coviu
- Doxy.me
- Facetime
- Facebook messenger
- Healthconnect
- HealthBank
- Vidyo
- WhatsApp
- Not applicable
- Other (please specify)

What device/s did/do your clients use to attend their Exercise Physiology sessions via Telehealth (more than one entry accepted)?

- Mobile Phone
- Tablet
- Laptop/PC
- Not applicable
- Email
- Other (please specify)

What is the usual fee and duration for face-to-face consultations with you?

- Usual fee ($)
- Usual duration (minutes)

What is the usual fee and duration for telehealth consultations with you?

- Usual fee ($)
- Usual duration (minutes)

What is your opinion with regards to the business costs of offering individual consultations via video over the internet versus in-person consultations?

- Consultations via video over the internet would cost the business more than in-person consultations
- Both consultations via video over the internet and in-person consultations would be equal
- Consultations via video over the internet would cost the business less than in-person consultations
- I don’t know
- Not applicable

Do you use any additional resources to support your consultations? (select all that apply)

- Text message reminders
- Follow up phone calls
- Apps for a smart phone or tablet
- Educational material about the issue/condition
- Written instructions, diagrams or booklets
- Videos
- Suggested websites for further information
- Logbooks and diaries
- Provision/purchase of equipment or devices
- Other

In response to changes to clinical practice due to COVID-19, did you adopt/implement/increase use of telehealth to deliver exercise physiology services?

- Yes
- No

Please select the reason that best describes why you did not adopt/implement/increase use of telehealth to deliver exercise physiology services

- Client preference
- Client safety
- Unable to access a reliable platform
- Cost of set-up to AEP or AEP business
- Cost of set-up or access for client (including lack of funding)
- Lack of AEP knowledge in conducting Telehealth services
- Lack of AEP confidence in conducting Telehealth sessions
- Service not suited to client physical abilities
- Service not suited to client cognitive abilities
- Language or cultural barriers
- Other (please specify)

Prior to COVID-19, on average, how many clients would you see in a week face-to-face?

Prior to COVID-19, on average, how many hours per week would you spend delivering Exercise Physiology services face-to-face?

Prior to COVID-19, on average, how many hours per week would you spend delivering Exercise Physiology services using Telehealth?

After the 25th of January 2020, approximately how many clients did you offer to facilitate Exercise Physiology services to via Telehealth platforms? (Note: this includes offers you made that were not necessarily accepted by the client)

Of these offers, approximately how many clients did you facilitate Exercise Physiology services to via Telehealth platforms?

Please select the reason that you believe best describes your clients’ reluctance to accept Exercise Physiology services via Telehealth platforms (more than one entry accepted):

- Not applicable
- Client preference
- Client safety concerns
- Cost of set-up or access for client (incl. lack of funding)
- Unable to access a reliable platform
- Service not suited to client physical capabilities
- Language or cultural barriers
- Other (please specify)

After the 25th of January 2020, how many existing clients did you elect NOT to offer or facilitate Exercise Physiology services to via Telehealth platforms?

Please select the reason that best describes your reluctance to offer Exercise Physiology services via Telehealth platforms (more than one entry accepted):

- Not applicable
- Ceased or suspended all services
- Client safety concerns
- Cost of set-up or access for client (incl. lack of funding)
- Unable to access a reliable platform
- Service not suited to client physical capabilities
- Service not suited to client cognitive capabilities
- Language or cultural barriers
- Other (please specify)

Of the clients who consented to receive Exercise Physiology services via Telehealth platforms, what percentage of clients completed their full treatment protocol? (e.g. 80% = "80")

If any client did not complete FTF programs, please select the option/s that best describes the reason for non-completion of the prescribed Telehealth program (more than one entry accepted):

- Not applicable
- Client preference
- Client lack of confidence in services via Telehealth
- Client lack of understanding or knowledge in services via Telehealth
- Client lack of interest or importance in utilising services via Telehealth
- Client safety concerns
- Difficulties with access to a reliable platform
- Problems unrelated to service delivery
- Other (please specify)

Please select the option/s that best describes the reason for the non-completion of the face-to-face programs (more than one entry accepted):

- Not applicable
- Client preference
- Client lack of confidence in services face-to-face
- Client lack of understanding or knowledge in services face-to-face
- Client lack of interest or importance in utilising services face-to-face
- Client safety concerns
- Difficulties with access to service providers venue
- Problems unrelated to service delivery
- Other (please specify)

Since Jan 25th, 2020, were there any serious adverse events in your patients using Telehealth services? (Note: A “serious” adverse event refers to a reaction to therapy that results in death, or is life threatening and/or requires hospitalisation.)

- No
- Yes

If yes, what was the nature of the serious adverse event?

If yes, was this event related directly to the Exercise Physiology service?

- Yes
- No

Since Jan 25th, 2020 were there any serious adverse events in your patients using face-to-face services?(Note: A “serious” adverse event refers to a reaction to therapy that results in death, or is life threatening and/or requires hospitalisation.)

- No
- Yes

If yes, what was the nature of the serious adverse event?

If yes, was this event related directly to the Exercise Physiology service?

- Yes
- No

Since Jan 25th, 2020 were there any adverse events in your patients using Telehealth services? (Note: An “adverse event” refers to any untoward medical occurrence associated with a therapy in humans, whether or not considered related to the therapy itself)

- No
- Yes

If yes, what was the nature of the adverse event?

If yes, was this event related directly to the Exercise Physiology service?

- Yes
- No

Since Jan 25th, 2020 were there any adverse events in your patients using face-to-face services? (Note: An “adverse event” refers to any untoward medical occurrence associated with a therapy in humans, whether or not considered related to the therapy itself)

- No
- Yes

If yes, what was the nature of the adverse event?

If yes, was this event related directly to the Exercise Physiology service?

- Yes
- No
